# Nutrition transition: contextualising the prevalence of overweight and obesity among the Lotha Naga tribal population of North-East India

**DOI:** 10.3389/fpubh.2026.1819420

**Published:** 2026-05-07

**Authors:** Jenny Jami

**Affiliations:** Department of Anthropology, North-Eastern Hill University, Shillong, India

**Keywords:** central obesity, nutrition transition, overweight and obesity, processed food, sedentary, traditional, tribal

## Abstract

**Background:**

The global rise in overweight and obesity has become a critical public health concern, increasingly affecting low and middle-income countries such as India, where a dual burden of undernutrition and overnutrition now coexists. Within this broader nutrition transition, tribal communities—including the Lotha Nagas—are experiencing rapid dietary and lifestyle changes linked to socioeconomic transformation, highlighting the need for more research in this area.

**Methods:**

A community-based cross-sectional study was conducted among 708 adult Lotha Nagas in Wokha Town, Nagaland. Data on socio-demographic characteristics, occupation, dietary habits (7-day FFQ), and physical activity (IPAQ) were collected through face-to-face interviews, and standardised anthropometric measurements (height, weight, waist circumference) were obtained to assess BMI (WHO Asian cut-offs) and central obesity (South Asian waist circumference criteria). Data were analysed using SPSS, applying descriptive statistics and chi-square–based tests, with statistical significance set at *p* < 0.05.

**Results:**

Traditional rice-based diets continue among the Lotha Nagas, but market-based and industrially manufactured foods are increasingly consumed, particularly by younger and socio-economically mobile groups. However, links between specific food items and overweight/obesity were inconsistent, indicating a multifactorial pattern. In contrast, the shift from agrarian to more sedentary occupations—especially among government employees and homemakers—was more clearly associated with overall and central obesity. Age and marital status showed more consistent relationships with nutritional outcomes.

**Conclusion:**

Overweight and obesity among the Lotha Nagas reflect an ongoing nutrition and lifestyle transition characterised by reduced physical activity, occupational shifts away from agriculture, and increasing incorporation of market-based foods. In line with the nutrition transition framework, these findings highlight the need to interpret changing body composition within broader socio-economic and cultural transformations.

## 1 Introduction

The concept of nutrition transition, first systematically articulated by Barry M. Popkin in the early 1990s, describes the shift away from traditional, fibre-rich diets and physically active lifestyles toward increased consumption of fats, sugars, and processed foods, driven by urbanisation, economic development, and globalization (1–7). The most consequential epidemiological manifestations of nutrition transition are the rising prevalences of overweight and obesity, alongside the persistence of undernutrition. Globally, overweight and obesity have reached pandemic proportions, with global estimates further projected to increase exponentially over the years (8, 9).

Obesity is a well-established major risk factor for a broad spectrum of non-communicable diseases (NCDs), including type 2 diabetes mellitus, cardiovascular diseases, certain cancers, musculoskeletal disorders, and metabolic syndrome (10–12) and its epidemiological burden—including premature mortality, reduced life expectancy, escalating healthcare costs, and lost economic productivity—is substantial (12–15, 58). Critically, this burden falls disproportionately on lower-income countries, where obesity prevalence is rising most rapidly, even as food insecurity and micronutrient deficiency remain unresolved (10, 13, 16–18). The World Obesity Federation (9) projects that nine of the ten countries facing the greatest increases in obesity—among both adults and children—are low or lower-middle-income nations in Asia or Africa, where health systems are least equipped to manage a growing NCD burden (9). These nations consequently face the double burden of malnutrition (DBM), wherein undernutrition in the form of stunting, wasting, underweight, and micronutrient deficiencies coexists paradoxically with escalating overweight, obesity, and diet-related NCDs (57, 59, 60).

India, a developing nation, exemplifies this transition with particular acuity, increasingly prevalent even among individuals with normal BMI in South Asian and tribal populations, independently confers heightened cardiometabolic risk comparable to generalised obesity (19–22), underscoring the limitations of BMI alone as a universal screening tool and the need for population-specific anthropometric assessments in nutritional research involving these communities.

Despite the accelerating pace of nutritional change among India’s tribal populations, empirical research on nutrition transition in this context remains limited, and the tribes of Northeast India are particularly underrepresented in the existing literature. The present study addresses this gap by focusing on the Lotha Nagas of Nagaland, with the primary objective of situating the high prevalence of overweight, obesity, and central obesity documented in this community (23) within the framework of nutrition transition. Specifically, through an integration of anthropological perspectives and quantitative analysis, the study aims to identify and examine the dietary patterns, physical activity patterns and socio-cultural factors driving nutritional change among the Lotha Nagas with the broader goal of generating locally grounded, culturally sensitive evidence relevant to public health policy for tribal populations at comparable stages of nutrition transition.

## 2 Materials and methods

### 2.1 Study design and setting

A community-based cross-sectional study was conducted among the Lotha Naga tribal population in Wokha town, Nagaland, between May 2014 and January 2016. Wokha Town, the only statutory urban centre in the district and the homeland of the Lotha Nagas, was purposively selected to examine nutritional changes in an urban tribal setting.

### 2.2 Study population

The study included adult Lotha Naga men and women aged ≥20 years. Pregnant women and individuals with conditions affecting accurate anthropometric measurement were excluded.

### 2.3 Sampling procedure

A two-way cluster sampling method was adopted. Five wards were randomly selected as the primary sampling units, and eligible individuals within these wards constituted secondary units. In the absence of a sampling frame, house-to-house visits were conducted, and all consenting adults aged 20–70 years were included. The sample was gender-stratified and comprised of 708 participants (354 men and 354 women) from 470 households.

Data on socio-demographic characteristics, occupation, dietary habits, and physical activity were collected using a pre-tested, semi-structured interview schedule administered through face-to-face interviews. Dietary habits were explored through questions on the frequency of consumption of traditional foods and market-based processed foods using a food frequency questionnaire/FFQ (24) based on a recall period of 7 days before the survey. Physical activity was measured using the International Physical Activity Questionnaire (IPAQ), and metabolic equivalent (MET) scores were computed.

### 2.4 Nutritional status assessment

Standardized procedures were used to collect anthropometric measurements: height was recorded to the nearest 0.1 cm with an anthropometer, weight was recorded to the nearest 0.5 kg using a portable weighing scale, and waist circumference was measured to the nearest 0.1 cm with a measuring tape (25–27). Nutritional status was evaluated using Body Mass Index (BMI), calculated as weight (kg)/height (m^2^), and classified according to WHO Asian cut-offs (28–30): <18.5 kg/m^2^ (underweight), 18.5–22.9 kg/m^2^ (normal), 23.0–24.9 kg/m^2^ (overweight), and ≥25.0 kg/m^2^ (obese). Central obesity was defined using waist circumference (WC) cut-offs recommended for South Asians (27, 31): ≥90 cm for men; ≥80 cm for women.

### 2.5 Ethical considerations

Ethical approval was obtained from the Institutional Ethics Committee for Human Samples/Participants (IECHSP) of the North-Eastern Hill University. Participation was voluntary, and confidentiality was maintained.

### 2.6 Data analysis

Data were entered and analyzed using SPSS for Windows. Descriptive statistics are presented as frequencies and percentages. Associations between variables were examined using Pearson’s chi-square test, Somers’ *d*, and the Cochran–Armitage test for trend. Effect sizes for chi-square tests were assessed using Phi (*ϕ*) and Cramer’s V (32). Statistical significance was set at *p* < 0.05.

## 3 Results

### 3.1 Socio-demographic characteristics of the participants

Socio-demographic variables included age, gender, place of birth, marital status, family size, education, income, and occupation. Gender was classified as men and women; place of birth as urban or rural; and marital status as unmarried, married, or divorced/separated/widowed. Family size was grouped into small (≤4 members), medium (16, 25), and large (≥7). Education was categorised into illiterate, primary, secondary, higher/senior secondary, and undergraduate and above, following the Indian Standard Classification of Education (33) with minor modifications. Per capita monthly income was derived from total household income and classified into quartiles: low (<₹2,500), lower middle (₹2,500–4,000), upper middle (₹4,000–6,295), and high-income groups (>₹6,295). Occupation was grouped into eight categories: government employee, non-government salaried employee, self-employed, agriculturist/labourer, student, homemaker, retired, and unemployed.

The final sample comprised 708 adult Lotha Nagas (354 men and 354 women) residing in Wokha Town, with a mean age of 37.64 ± 13.89 years. Most participants were born in rural areas (63.28%), and 63.70% were married. Medium family size (5–6 members) was most common (40.25%), followed by small (32.20%) and large families (27.54%). Secondary education was the predominant educational level (35.45%). The largest proportion of participants belonged to the upper middle-income group (29.38%). Government employees (25.85%) and homemakers (22.60%) constituted the major occupational groups. Detailed descriptive statistics are provided in Supplementary Sheet S1.

### 3.2 Food habits: from traditional to market-based diets

Early ethnographic accounts describe the traditional Lotha diet as diverse, largely subsistence-based and closely tied to the ecological environment. J. P. Mills (34), in The Lhota Nagas, noted that the diet included domesticated and wild meats, fish, insects, cultivated crops, and forest produce. Similarly, Myanbemo (35) observed although daily meals were simple—primarily rice, vegetables, bamboo shoots, and dry fish—the Lothas consumed a wide variety of available foods in small quantities. Rice has historically been the staple, accompanied by leafy vegetables (both wild and home-grown), roots and tubers (notably yam), pulses, and chilli-based preparations. Fermented and dried bamboo shoots formed essential condiments, reflecting the integration of food and cultural identity.

Animal-source foods have long occupied an important place in Lotha food culture. Pork, in particular, holds socio-cultural, ritual, and economic significance, frequently associated with customary feasts, exchanges, and ceremonial obligations (35, 36). Hunting and fishing, though reduced due to environmental and socio-economic changes, historically contributed to dietary diversity (37). In contrast, milk consumption was minimal (38), as milking cattle was not customary (34). Traditional rice beer (*zütsü*) once played a central role in ritual and social life but declined considerably following the spread of Christianity.

Ethnographic observations from the present study indicate that while traditional food preferences persist, substantial changes have occurred in food procurement and consumption patterns. Increased reliance on market supply has altered dietary composition and frequency. Contemporary food habits in the present study were assessed based on the frequency of food consumption under major food categories (39, 24) and the intake of industrially manufactured food products. Detailed descriptive statistics are provided in Supplementary Sheet S2. The study shows universal daily consumption of rice and a high daily intake of leafy vegetables (80.08%) among the Lotha Nagas. Roots and tubers are consumed daily (51.13%) or weekly (40.68%), whereas fruits are consumed mainly occasionally (63.70%). Animal-source foods such as fish, poultry, eggs, and meat are generally consumed weekly or occasionally; however, most participants (64.41%) reported eating meat at least once a week, with significantly higher intake among men (*χ^2^* = 16.51, *p* < 0.001, Cramer’s *V* = 0.15).

Fresh milk consumption remains rare (80.93% reporting never or rare use of fresh milk), though powdered milk is commonly added to tea or coffee, which are consumed daily by 86.58% of participants. In addition to powdered milk, other market-based foods are increasingly incorporated into the diet. A notable proportion reported occasional consumption of carbonated beverages (37.71%), with significant associations observed across gender (*χ^2^* = 13.57, df = 3, *p* = 0.004), age (*χ^2^* = 82.55, df = 12, *p* < 0.0005), place of birth (*χ^2^* = 27.50, df = 3, *p* < 0.0005), marital status (*χ^2^* = 11.98, df = 6, *p* < 0.0005), education (*χ^2^* = 37.42, df = 12, *p* < 0.0005), and occupation (*χ^2^* = 73.02, df = 21, *p* < 0.0005). Younger adults (20–29 years), unmarried individuals, and students demonstrated higher intake. Similar socio-demographic patterns were observed for sweet products and packaged processed foods. Intake of sweet products was found to be associated with age (*χ^2^* = 64.77, *df* = 12, *p* < 0.0005), marital status (*χ^2^* = 45.92, df = 6, *p* < 0.0005), education (*χ^2^* = 28.60, df = 12, *p* = 0.005), and occupation (*χ^2^* = 37.01, df = 21, *p* = 0.02). Approximately 31.07% reported daily consumption of packaged processed foods, with women reporting significantly higher intake than men (*χ^2^* = 12.88, *p* = 0.005, Cramer’s V = 0.14). Although fast-food consumption remains relatively infrequent, it shows a statistically significant association with all the socio-demographic variables examined. Higher intake is observed among younger, more educated, and higher-income groups, suggesting the gradual diffusion of globalised food systems. Descriptive statistics of these associations are provided in Supplementary Sheet S3.

Changes in culinary practices and daily meal structures among the Lothas further reflect the broader processes of dietary transition. Traditionally, boiling was the predominant method of preparation (34, 35), with minimal use of cooking oils. The present study indicates an increased use of commercially available cooking oils, though mostly on an occasional (50.85%) or weekly (34.32%) basis.

These changes are further evident in evolving meal patterns. Historically, boiled rice accompanied by vegetables, bamboo shoots, dry fish, and occasionally meat was consumed three times daily—early morning meal (*enyathüng-etso*), noon meal (*nshi*), and evening meal (*mmyu-etso*)—with similar food items served across meals (34, 35, 38). While traditional meal timings and rice-based patterns largely persist, notable changes have emerged in portion sizes and food choices. Urban residents increasingly begin the day with tea and snack foods alongside the customary rice meal, often consume lighter snack-based midday meals, and continue with a substantial rice-based dinner at sunset. Thus, although the foundational dietary framework remains intact, the integration of tea and energy-dense snack items—especially during breakfast and midday meals—marks a transition in food preferences. Importantly, this dietary shift coincides with reduced physical activity associated with occupational and lifestyle changes, potentially contributing to an imbalance between energy intake and expenditure.

**Figure 1:**
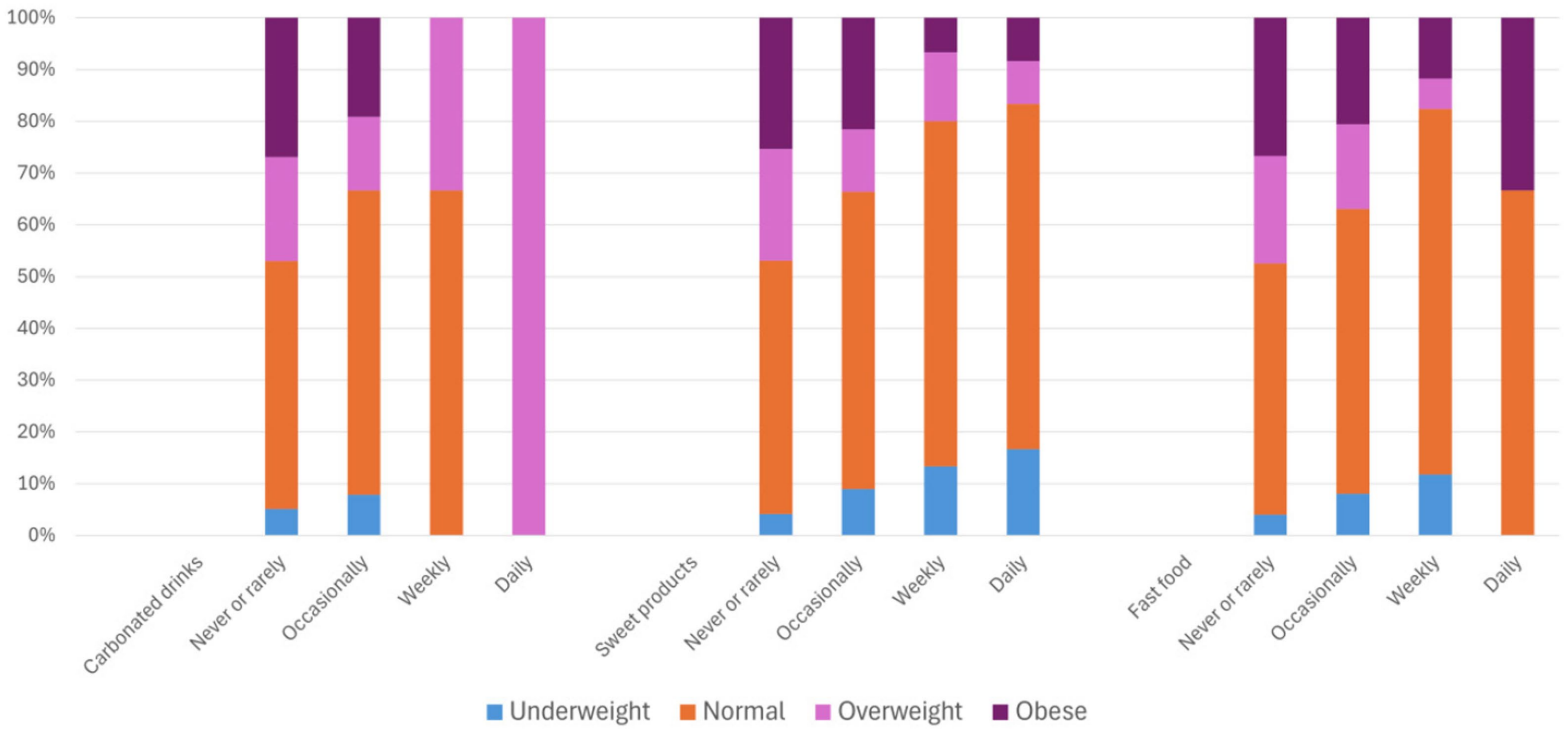
Descriptive statistics of nutritional status (based on BMI Asian criteria) by selected food habits.

**Figure 2:**
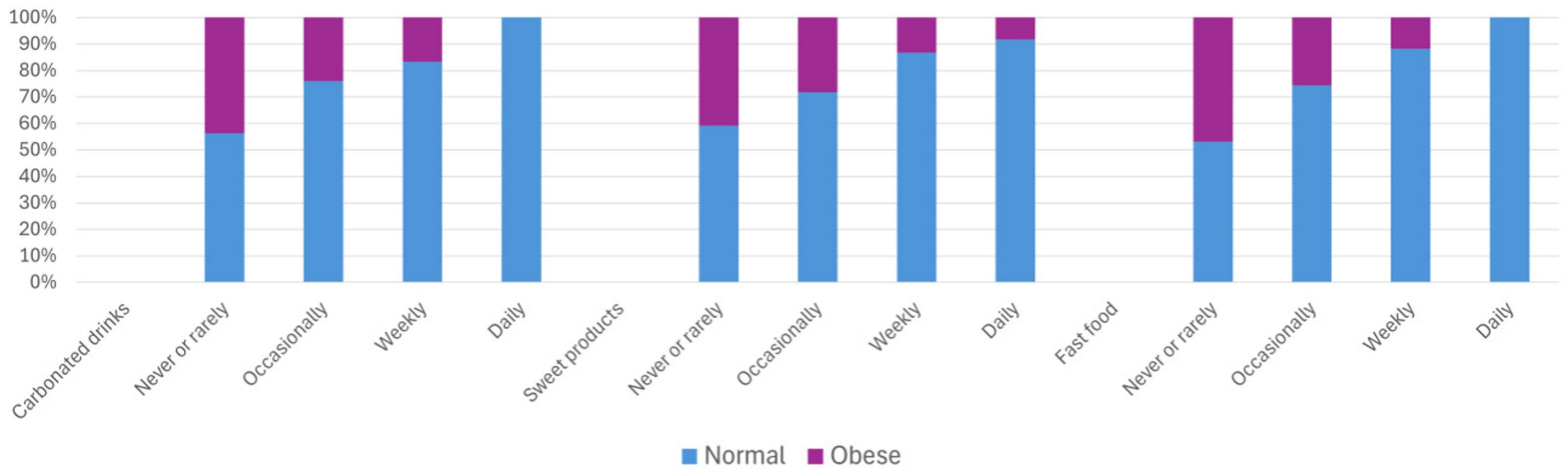
Descriptive statistics of central obesity (based on WC) by selected food habits.

Statistical analyses suggest that the relationship between food habits and nutritional outcomes is complex (Supplementary Sheets S4, S5). Somers’*d* indicated no significant associations between overweight/obesity and consumption of cooking oils, tea/coffee, or processed foods. Although statistically significant associations were observed for carbonated beverages (*d* = −0.14, *p* < 0.0005), sweet products (*d* = −0.16, p < 0.0005), and fast food (*d* = − 0.13, p < 0.0005), the patterns were inconsistent and did not clearly explain the prevalence of overweight or obesity (Figure 1). The Cochran–Armitage test indicated significant associations between central obesity and intake of carbonated drinks (*χ^2^* = 30.25, df = 3, *p* < 0.0005), sweet products (*χ^2^* = 17.82, df = 3, *p* < 0.0005), and fast food (*χ^2^* = 39.99, df = 3, *p* = 0.0001); however, these relationships remain inconclusive (Figure 2). The use of broad frequency categories limits interpretability; moreover, the observed consumption patterns may be confounded by socio-economic determinants of food access, choice, and procurement, as well as by other unmeasured variables, as previously discussed. In general, processed foods in the form of carbonated drinks, sweet products and fast food were consumed more frequently among the younger age groups, whereas individuals in older age groups reported rarely or never consuming such items, suggesting that the prevalence of overweight and obesity among the older adults in the present study may be explained by other factors, especially the socio-demographic variables and physical activity levels.

### 3.3 Physical activity: transition in energy expenditure

Traditionally, the Lotha Naga economy was agrarian, centred on jhum (shifting cultivation), with both men and women engaged in physically demanding agricultural labour and allied handicraft activities. Although agriculture remains important in the district, accounting for.60.54% of the working population—urban occupational patterns show substantial diversification. Census data indicate that only 9.42% of the urban workforce is engaged in agriculture, while the majority are classified as other workers (40, 41). A similar pattern emerged in the present study, where only 5.37% of participants were cultivators or labourers. Most respondents were employed in government service (25.85%), were homemakers (22.60%), self-employed (13.42%), or students (12.01%), with smaller proportions in non-government salaried employment, unemployment, or retirement. These largely sedentary occupations represent a marked shift from traditional physically intensive livelihoods.

**Figure 3:**
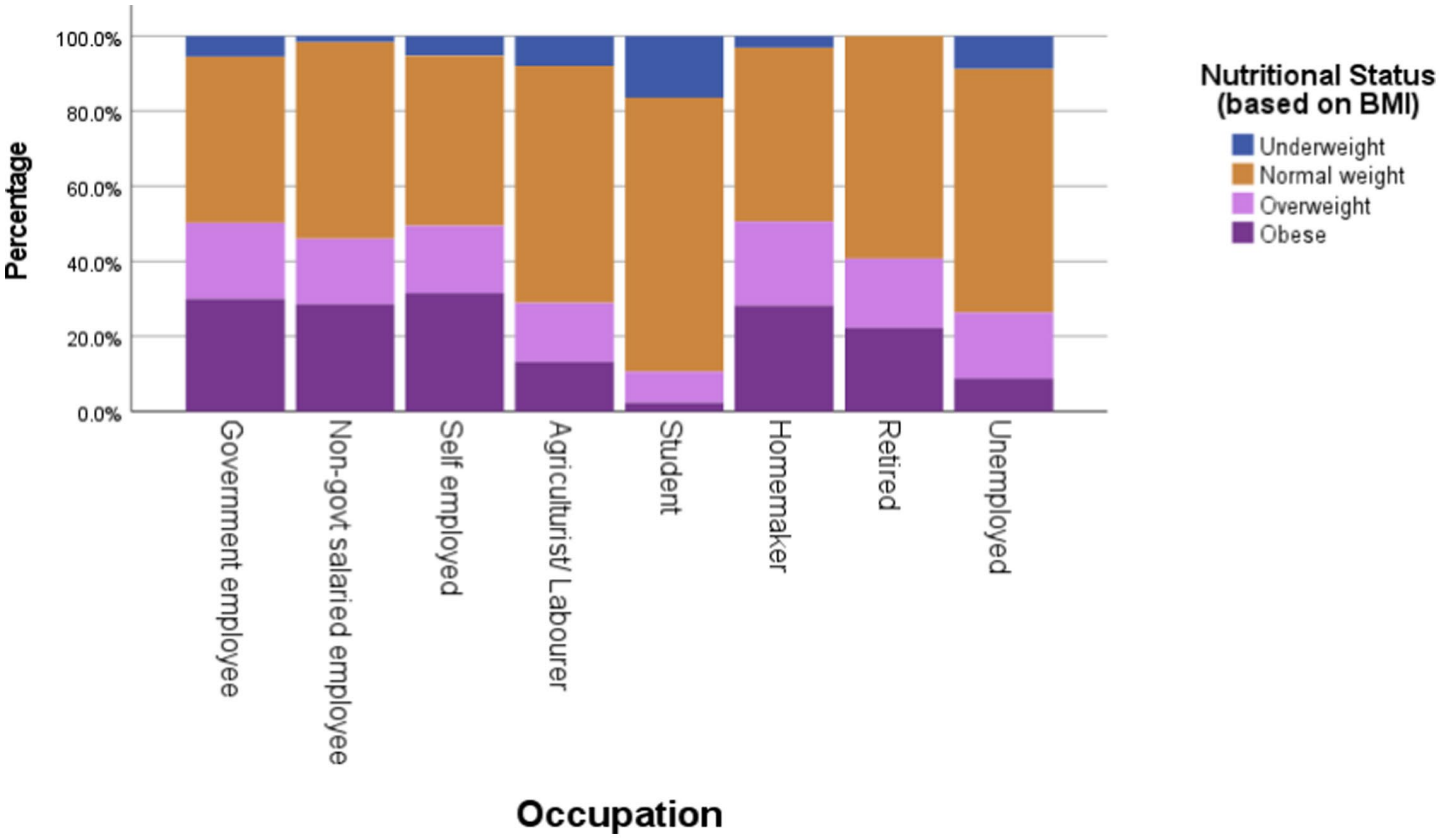
Descriptive statistics of nutritional status (based on BMI) by occupation.

**Figure 4:**
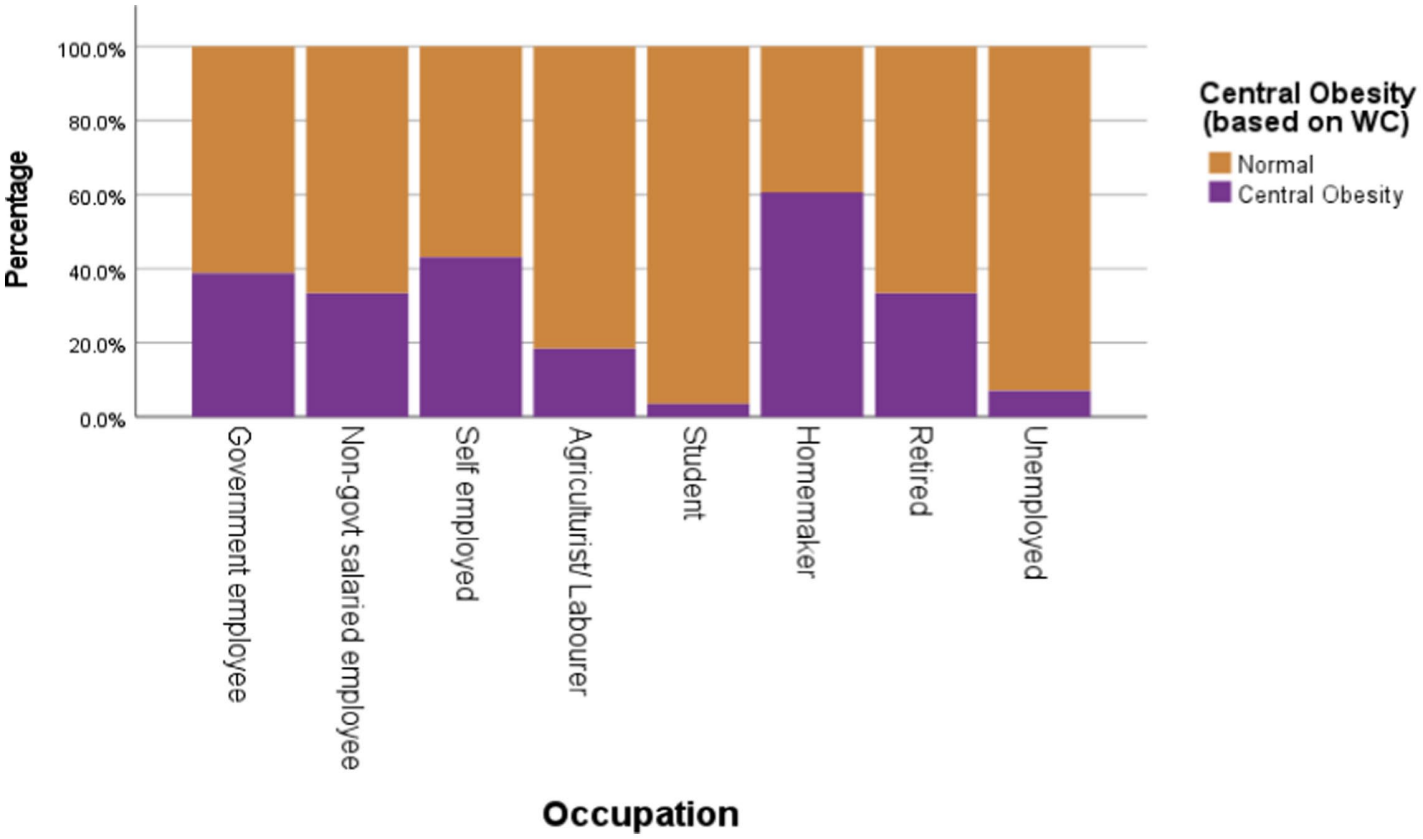
Descriptive statistics of central obesity (based on WC cut-offs) by occupation.

**Figure 5:**
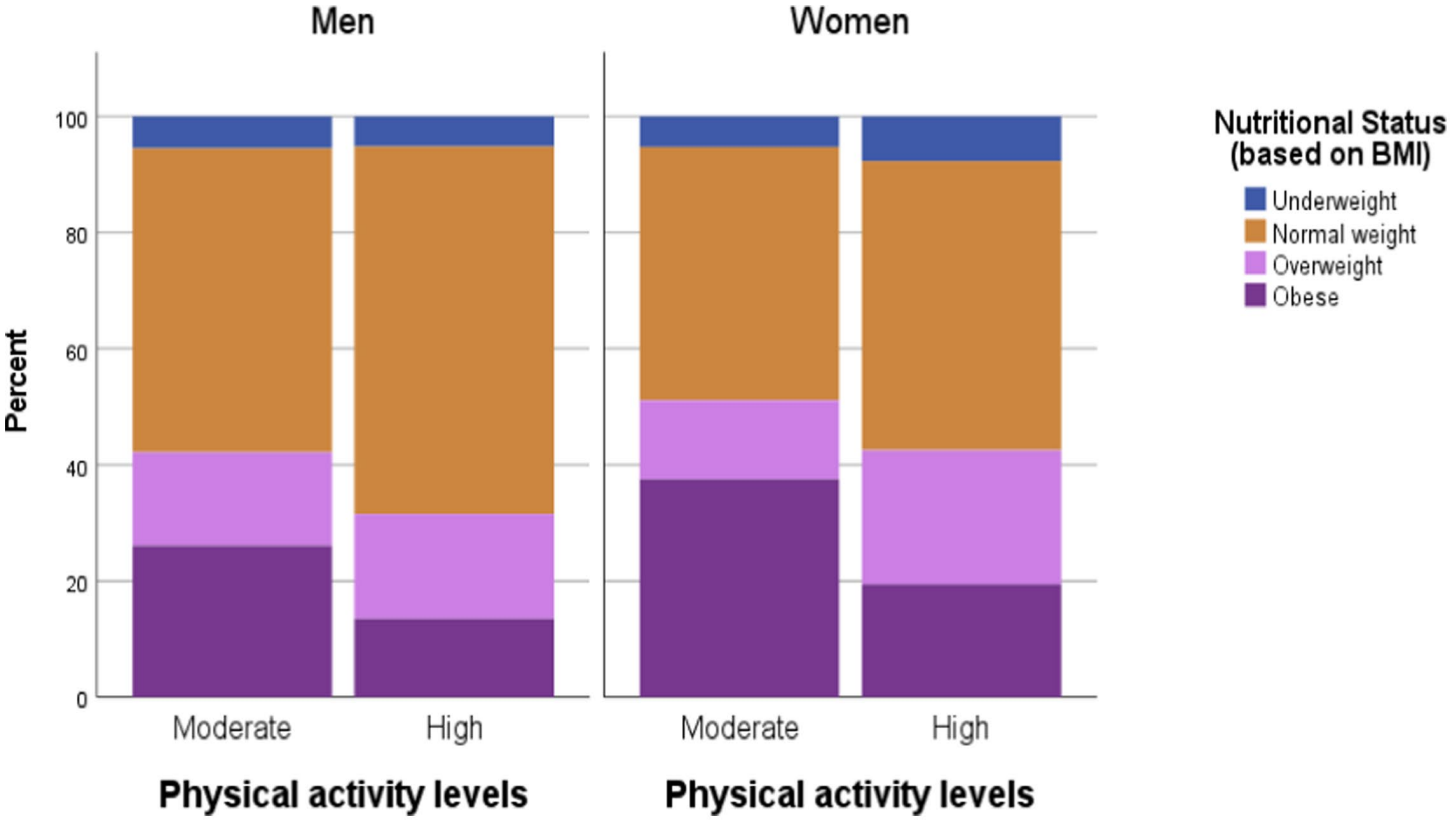
Descriptive statistics of nutritional status (based on BMI) by Physical activity.

**Figure 6:**
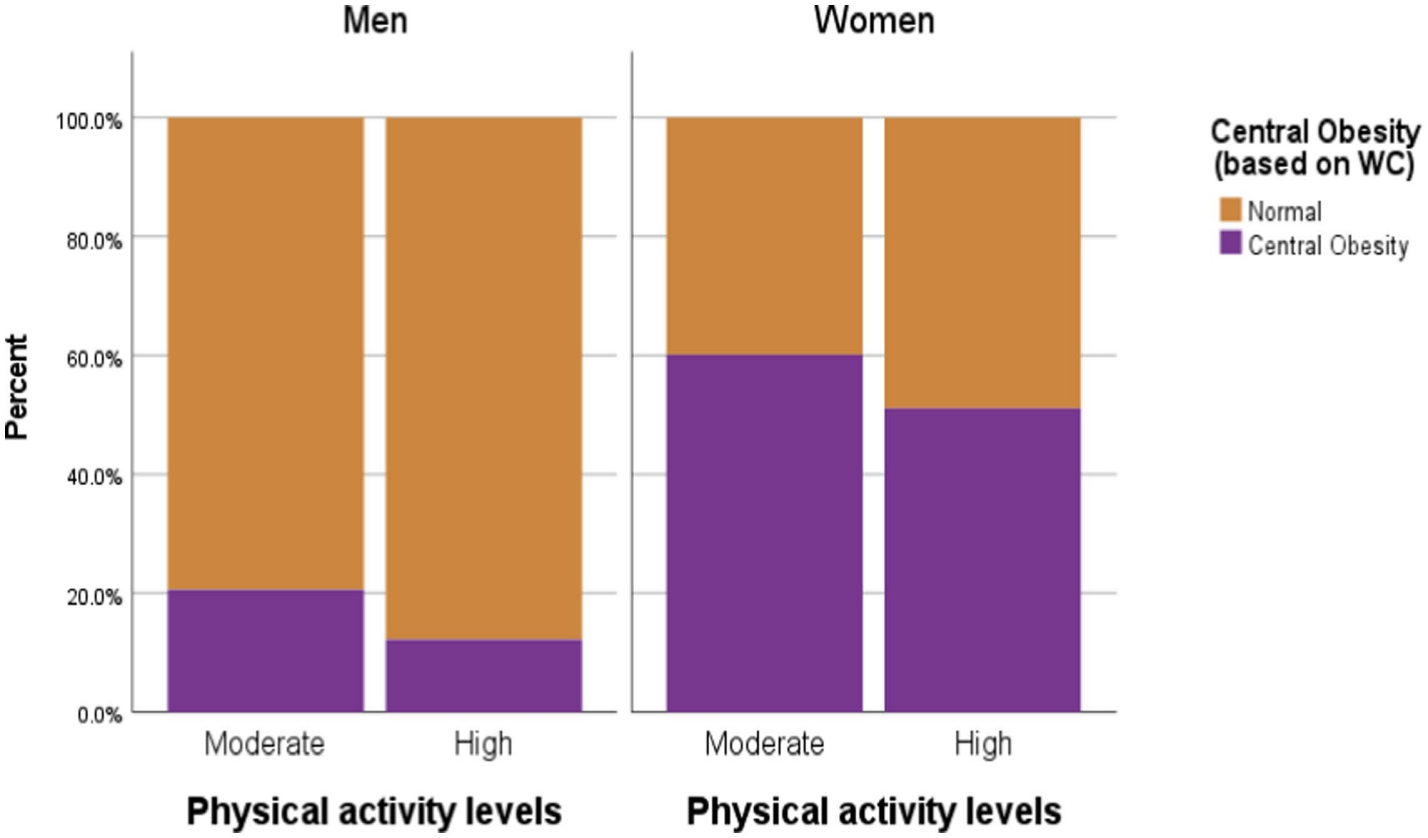
Descriptive statistics of central obesity (based on WC) by Physical activity.

Occupation was significantly associated with both overall nutritional status (*χ^2^* = 73.66, df = 7, *p* < 0.0005) and central obesity (*χ^2^* = 74.52, df = 21, *p* < 0.0005). The combined prevalence of overweight and obesity was highest among homemakers (50.63%) and government employees (50.27%) and lowest among students (10.59%), as presented in Figure 3 and Figure 4 numbers in the low-activity category, participants were grouped into moderate and high physical activity levels for analysis. Overweight/obesity and central obesity were present in both categories, but were more prevalent among those reporting moderate activity levels (Figure 5 and Figure 6).

Chi-square analysis showed a significant association between physical activity and nutritional status in both men (*χ^2^* = 8.75, *p* = 0.03, Cramer’s *V* = 0.16) and women (*χ^2^* = 15.66, *p* = 0.001, Cramer’s *V* = 0.21). Central obesity was also significantly associated with physical activity among men (*χ^2^* = 4.31, *p* = 0.04, *ϕ* = −0.11), though not among women. Overall, the findings suggest a transition toward reduced energy expenditure linked to occupational and lifestyle changes in the urban setting.

### 3.4 Role of socio-demographic characteristics

The present study examined the relationship between nutritional status and socio-demographic factors, including gender, age, place of birth, family size, marital status, education, income, and occupation (Supplementary Sheet S6). Nutritional status was not significantly associated with gender (*χ^2^* = 6.74, df = 3, *p* < 0.08) or family size (*χ^2^* = 5.86, df = 6, *p* < 0.44). However, statistically significant associations were observed with age group (*χ^2^* = 1.11, df = 12, *p* < 0.0005), place of birth (*χ^2^* = 24.52, df = 3, *p* < 0.0005), marital status (*χ^2^* = 83.28, df = 6, *p* < 0.0005), education (*χ^2^* = 33.89, df = 12, *p* = 0.001), income (*χ^2^* = 17.49, df = 9, *p* < 0.04), and occupation (*χ^2^* = 74.52, df = 21, *p* < 0.0005). The strength of association was comparatively higher for age (Cramer’s *V* = 0.23) and marital status (Cramer’s *V* = 0.24), and smaller for place of birth (0.19), occupation (0.19), education (0.13), and income (0.09).

Except for family size (*χ^2^* = 0.75, df = 2, *p* < 0.69), central obesity was significantly associated with all socio-demographic variables, i.e., gender (*χ^2^* = 1.09, df = 1, *p* < 0.0005), age groups (*χ^2^* = 1.39, df = 4 *p* < 0.0005), place of birth (*χ^2^* = 17.77, df = 1, *p* < 0.0005), marital status (*χ^2^* = 1.36, df = 2, *p* < 0.0005), education (*χ^2^* = 55.19, df = 4, *p* < 0.0005), income (*χ^2^* = 8.94, df = 3, *p* = 0.03), and occupation (*χ^2^* = 1.10, df = 7, *p* < 0.0005). The associations were moderate for gender (*ϕ* = 0.39), age (Cramer’s V = 0.44), marital status (0.44), occupation (0.40), and education (0.28), and weaker for place of birth (ϕ = 0.16) and income (0.11).

### 3.5 Major factors associated with the prevalence of overweight and obesity

The above findings indicate that nutritional status is associated with most of the variables under study. Accordingly, logistic regression analysis was computed to understand the effect of these variables on nutritional status. The variables included in the regression model were gender, age, place of birth, marital status, education, income, occupation and physical activity. The categorization of these variables remained consistent with that described in the methodology section, except for marital status, which was reclassified into two categories: ever-married and unmarried. The ever-married category comprised individuals who had been married at least once in their lifetime, regardless of their current marital status. Accordingly, respondents who were divorced, separated, or widowed were also clubbed in this category for statistical analysis. For the outcome variable, overweight or obesity was combined into a single category. The prevalence was determined using the BMI classification applied in the present study (BMI ≥ 23 kg/m^2^). Central obesity was assessed based on waist circumference measurements.

A binomial logistic regression was conducted to examine the effects of age, place of birth, marital status, education, income, and physical activity on the prevalence of overweight or obesity. The overall model was statistically significant, *χ^2^* (20) = 140.12, *p* < 0.0005, indicating that the predictors collectively distinguished between individuals who were overweight/obese and those who were not. The Hosmer and Lemeshow goodness-of-fit test was not statistically significant (*p* = 0.174), suggesting that the model adequately fit the data. The model accounted for 24.20% (Nagelkerke *R^2^*) of the variance in overweight or obesity and correctly classified 68.40% of cases. The sensitivity of the model was 60.00%, while specificity was 74.30%. The positive predictive value was 62.54% and negative predictive value was 72.23%. The odds ratio derived from the logistic regression are presented in Table 1. Among the variables included in the model, age, marital status and physical activity showed statistically significant associations with overweight or obesity. Compared to adults agede 20–29 years (reference category), those aged 30–39 years had 1.75 times higher odds (95% CI: 1.01–3.02) of being overweight or obese. Adults aged 40–49 years and 50–59 years had 3.17 (95% CI: 1.75–5.76) and 2.99 (95% CI: 1.56–5.76) times higher odds, respectively, of being overweight or obese than those in the reference group. Similarly, individuals aged 60–70 years had 1.12 times higher odds (95% CI: 1.12–5.43) of being overweight or obese compared to adults aged 20–29 years. Married individuals were 2.53 times more likely (95% CI: 1.36–4.69) to be overweight or obese than unmarried individuals. In terms of physical activity, adults with moderate levels of physical activity had 1.91 times higher odds (95% CI: 1.34–2.75) of being overweight or obese compared to those who were highly active.

**Table 1:** Odds ratio derived from logistic regression for the risk factors of overweight or obesity (based on BMI).

Model*	Estimate	*SE*	Wald	*p-*value	Odds ratio (*OR*) exp. B	95% CI for exp. (B)	
						Lower	Upper
Age Groups (years)			17.075	**0.002**			
20–29	Reference						
30–39	0.560	0.278	4.062	**0.044**	1.750	1.015	3.016
40–49	1.155	0.304	14.449	**<0.001**	3.174	1.750	5.758
50–59	1.096	0.324	10.788	**0.001**	2.993	1.556	5.758
60–70	0.903	0.402	5.041	**0.025**	2.467	1.122	5.427
Place of birth (urban as reference)	0.096	0.204	0.221	0.638	1.101	0.738	1.642
Marital status (unmarried as reference)	0.928	0.315	8.654	**0.003**	2.529	1.363	4.693
Education			2.873	0.412			
≤Primary	0.253	0.320	0.625	0.429	1.288	0.687	2.414
Secondary	−0.042	0.279	0.022	0.882	0.959	0.555	1.658
Higher secondary	−0.205	0.285	0.519	0.471	0.814	0.465	1.424
≥Undergraduate	Reference						
Income			5.904	0.116			
LIG	Reference						
MIG_L_	0.174	0.264	0.433	0.511	1.189	0.709	1.995
MIG_H_	0.502	0.256	3.843	**0.050**	1.652	1.000	2.730
HIG	0.560	0.280	3.995	**0.046**	1.750	1.011	3.030
Occupation			8.789	0.268			
Government employee	0.638	0.466	1.877	0.171	1.892	0.760	4.713
Non-govt. salaried employee	0.773	0.507	2.329	0.127	2.166	0.803	5.847
Self employed	0.642	0.491	1.714	0.190	1.901	0.727	4.975
Homemaker	0.713	0.483	2.180	0.140	2.041	0.292	5.260
Retired	−0.048	0.631	0.006	0.940	0.953	0.277	3.286
Agriculturist/Labourer	0.025	0.593	0.002	0.966	1.026	0.321	3.279
Unemployed	0.939	0.484	3.768	0.052	2.558	0.991	6.603
Student	Reference						
Physical activity (high as reference)	0.650	0.184	12.412	**<0.001**	1.916	1.334	2.750

*All variables included in this regression model were those which were found to have statistically.

Significant association with nutritional status (based on BMI Asian criteria) in the present study. Significant *p*-value results are shown in bold.

Again, a binomial logistic regression was conducted to examine the effects of gender, age, place of birth, marital status, education, income, occupation, and physical activity on central obesity, as defined by waist circumference. The overall model was statistically significant, *χ^2^* (21) = 332.05, *p* < 0.001, indicating that the predictors jointly distinguished between individuals with and without central obesity. The Hosmer and Lemeshow goodness-of-fit test was not statistically significant (*p* = 0.725), suggesting that the model adequately fit the data. The model explained 51.40% of the variance in central obesity (Nagelkerke *R^2^*) and correctly classified 79.20% of cases. The sensitivity was 67.60%, and specificity was 85.70%. The positive predictive value was 72.46%, while the negative predictive value was 82.63%. The odds ratio derived from regression analysis are presented in Table 2. Gender, age, marital status, and physical activity demonstrated statistically significant associations with central obesity. Women had 11.09 times higher odds (95% CI: 6.229–19.76) of being centrally obese than men. Relative to adults aged 20–29 years (reference category), those aged 30–39 years had 3.96 times higher odds (95% CI: 2.00– 7.83) of central obesity. Adults aged 40–49 years and 50–59 years had 7.65 (95% CI: 3.68–15.93) and 9.49 (95% CI: 4.27–21.11) times higher odds, respectively, of being centrally obese compared to the reference group. Similarly, individuals aged 60–70 years had 5.77 times higher odds (95% CI: 2.23–14.94) of central obesity than those aged 20–29 years. Married individuals were 7.09 times more likely (95% CI: 2.80–17.93) to be centrally obese than unmarried individuals. Furthermore, adults with moderate levels of physical activity had 2.68 times higher odds (95% CI: 1.69–4.24) of central obesity than highly active adults.

**Table 2:** Odds ratio derived from logistic regression for the risk factors of central obesity (based on waist circumference).

Model*	Estimate	*SE*	Wald	*p* value	Odds ratio (*OR*) exp. B	95% CI for exp. (B)	
						Lower	Upper
Gender (men as reference)	2.406	0.294	66.776	**<0.001**	11.094	6.229	19.759
Age groups (years)			36.718	**<0.001**			
20–29	Reference						
30–39	1.376	0.348	15.619	**<0.001**	3.958	2.001	7.829
40–49	2.035	0.374	29.595	**<0.001**	7.654	3.676	15.934
50–59	2.251	0.408	30.493	**<0.001**	9.496	4.271	21.109
60–70	1.753	0.485	13.071	**<0.001**	5.774	2.232	14.938
Place of birth (urban as reference)	−0.245	0.254	0.927	0.336	0.783	0.476	1.288
Marital status (unmarried as reference)	1.959	0.473	17.110	<0.001	7.089	2.803	17.932
Education			3.502	0.320			
≤Primary	0.443	0.385	1.326	0.250	1.557	0.733	3.310
Secondary	0.198	0.349	0.324	0.569	1.220	0.616	2.415
Higher secondary	−0.189	0.364	0.269	0.604	0.828	0.406	1.689
≥Undergraduate	Reference						
Income			4.713	0.194			
LIG	Reference						
MIG_L_	0.057	0.317	0.032	0.858	1.059	0.568	1.972
MIG_H_	0.195	0.307	0.402	0.526	1.215	0.665	2.220
HIG	0.626	0.335	3.489	0.062	1.869	0.970	3.604
Occupation				0.308			
Government employee	0.767	0.775	0.978	0.323	2.153	0.471	9.834
Non-govt. salaried employee	0.729	0.813	0.804	0.370	2.073	0.421	10.200
Self employed	0.841	0.792	1.126	0.289	2.318	0.491	10.946
Homemaker	0.343	0.786	0.190	0.663	1.409	0.302	6.571
Retired	0.740	0.903	0.672	0.412	2.096	0.357	12.293
Agriculturist/Labourer	−0.670	0.918	0.532	0.466	0.512	0.085	3.096
Unemployed	0.435	0.912	0.228	0.633	1.546	0.259	9.225
Student	Reference						
Physical activity (high as reference)	0.985	0.235	17.624	**<0.001**	2.679	1.691	4.244

*All variables included in this regression model were those which were found to have statistically.

Significant association with central obesity (based on waist circumference) in the present study. Significant *p*-value results are shown in bold.

## 4 Discussion

The present study situates the high prevalence of overweight, obesity, and central obesity among the Lotha Nagas within the broader framework of nutrition transition. The findings indicate that this indigenous community, historically characterised by subsistence agriculture, physically demanding livelihoods, and a predominantly traditional diet, is undergoing marked dietary and occupational changes consistent with wider processes of urbanisation and market integration.

Ethnographic accounts on the Lotha Nagas (34, 35) describes a traditional diet centred on rice, leafy vegetables, bamboo shoots, roots and tubers, and locally sourced animal protein, with minimal use of cooking oil and negligible consumption of milk or sugary foods. The present findings suggest that while these traditional dietary elements remain foundational, there has been a clear shift in food procurement and consumption patterns. Increasing reliance on market-based foods—such as packaged snacks, carbonated beverages, sweet products, and fast foods—reflects growing exposure to commercial food systems. The coexistence of local food production and global food supply chains reflects a dual food system (42), however, the growing dominance of the industrial food system poses a risk of gradually displacing traditional food systems, with potentially adverse consequences for dietary quality and nutritional health.

Dietary analyses indicated that the intake of ultra-processed foods was particularly evident among younger adults, unmarried individuals, students, and those with higher education or income levels, consistent with broader evidence that younger and socio-economically mobile groups are often early adopters of energy-dense, processed foods (43). Of concern, however, is the persistence of overweight and obesity among older adults, despite their relatively low consumption of industrially manufactured/ processed diets. The growing preference for processed foods among younger populations represents a compounding risk, with the potential to amplify obesity prevalence as these cohorts age.

Occupational transition emerged as a critical factor. The shift from labour-intensive agriculture toward sedentary employment was significantly associated with overall nutritional status and central obesity, with the highest combined prevalence of overweight and obesity among homemakers and government employees. Although participants reported moderate to high physical activity levels based on IPAQ classifications, overweight and central obesity were more prevalent among those in the moderate activity group. This may reflect overestimation of physical activity, qualitative differences in activity intensity, or the influence of dietary factors that offset energy expenditure. The significant association between physical activity and nutritional status—particularly among men—further supports the importance of energy balance in shaping emerging obesity patterns. The persistence of central obesity even among individuals reporting higher activity levels, however, suggests that occupational sedentism and dietary energy density may interact in complex ways.

Statistical analyses of socio-demographic factors indicated more consistent relationships with nutritional outcomes. Nutritional status showed significant associations with age, place of birth, marital status, education, income, and occupation, but not with gender or family size, with the strongest association observed for age and marital status. Central obesity was significantly associated with all socio-demographic variables except family size, with moderate associations for gender, age, marital status, occupation, and education, and weaker associations for place of birth and income. Logistic regression identified age, marital status, and physical activity as significant predictors of overweight/obesity (variance explained: 24.2%), with risk peaking in the 40–59 age group and among married and moderately active individuals. For central obesity (variance explained: 51.4%), gender emerged as an additional key predictor—women had 11 times higher odds than men, and married individuals were 7 times more likely to be centrally obese than unmarried ones, with risk increasing further with age. The observed association between central obesity and gender may reflect differences in fat distribution patterns shaped by both biological and socio-cultural influences. Education and income displayed comparatively weaker associations with overall overweight/obesity, and were linked to certain dietary behaviours, indicating that socio-economic mobility may influence food choices. The concentration of excess weight among more affluent groups mirrors patterns observed elsewhere in India and other low and middle-income settings (44–46), where larger body size is sometimes symbolically associated with prosperity and social status, though the cultural meanings attached to body size among the Lothas require deeper ethnographic exploration.

The rising prevalence of overweight and obesity among the Lothas reflects broader nutrition transition processes, wherein dietary modernization and increased consumption of energy-dense foods have occurred alongside reduced physical activity and shifting socio-demographic dynamics—patterns similarly observed among other tribal populations in India (47–50) and elsewhere (51, 52).

While gaps in nutrition awareness have been noted (38, 40), attributing nutritional outcomes primarily to a lack of awareness risks oversimplifying what is, in essence, a far more complex and structurally embedded process. Food choices are not merely individual decisions; they are shaped by and respond to broader structural transformations, including market integration, changing livelihoods, and shifting cultural meanings of food. Effectively addressing these trends, therefore, requires moving beyond individual-level explanations toward a political economy (53, 54) and biocultural (55, 56) perspective—one that is supported by longitudinal and interdisciplinary research capable of informing contextually appropriate and sustainable public health interventions.

## 5 Limitations

The findings are predominantly descriptive with considerable reliance on interpreting Supplementary materials. The assessment of food habits and physical activity was based primarily on food frequency measures and self-reported activity schedules, which may be subject to reporting bias. The use of a 7-day food frequency questionnaire, while practical for population-level screening, may be inadequate to fully characterise habitual dietary patterns, particularly in the context of obesity research where day-to-day variability in food intake and portion estimation are critical considerations. Physical activity levels may have been overestimated, and qualitative differences in activity intensity were not fully captured, thus limiting the interpretation of physical activity data derived from the IPAQ. Additionally, dietary factors that may offset energy expenditure could not be adequately quantified. A more precise estimation of energy balance would have been possible through detailed dietary assessments and objective measurements of caloric intake and expenditure. The cross-sectional design, while suitable for assessing prevalence and associations, is insufficient to support causal or directional inferences—a particularly notable limitation in nutrition transition research, where dietary and lifestyle changes unfold over time. Longitudinal data would be necessary to draw more robust conclusions about the drivers of nutritional change among the Lothas. Furthermore, the study lacked multivariate analysis. It was also limited in its ability to comprehensively examine the cultural dimensions shaping food practices and body norms, complexities that are often difficult to capture within predominantly quantitative research designs, thereby constraining the integration of deeper socio-cultural insights into the analysis.

## 6 Conclusion

The findings of this study indicate that overweight, obesity and central obesity among the Lotha Nagas are embedded within an ongoing nutrition and lifestyle transition marked by occupational shifts, reduced physical activity, and increasing integration into market-based food systems. Traditional food practices and moderate physical activity persist, yet they increasingly coexist with processed, commercially available foods and more sedentary occupational roles, signaling a gradual decline in overall energy expenditure. The relationships between food habits and nutritional outcomes were not uniformly linear, highlighting the multifactorial nature of overweight and obesity, while the convergence of socio-demographic factors—including age, gender, and marital status—with dietary and occupational change further underscores its complexity. Although the cross-sectional design limits causal interpretation, the patterns observed are consistent with the broader framework of nutrition transition, wherein dietary change, market integration, and livelihood change collectively shape emerging health risks—trends similarly documented among indigenous and tribal communities elsewhere. The present study on the Lotha Nagas illustrates the context-specific dynamics through which global forces of nutritional change intersect with local realities. Effectively addressing these trends demands more than awareness-based interventions; it requires culturally grounded, structurally informed, and longitudinally sustained public health strategies that recognise both the persistence of traditional practices and the growing influence of market-driven food systems, and that are guided by the political, economic, and biocultural dimensions of health in transitioning societies.

## Data Availability

The original contributions presented in the study are included in the article/Supplementary material, further inquiries can be directed to the corresponding author.
